# Voluminous Myoepithelioma of the Minor Salivary Glands Involving the Base of the Tongue

**DOI:** 10.1155/2016/3785979

**Published:** 2016-03-13

**Authors:** Mario Policarpo, Valentina Longoni, Pietro Garofalo, Paolo Spina, Francesco Pia

**Affiliations:** ^1^ENT Department, University of Eastern Piedmont, 28100 Novara, Italy; ^2^Pathology Department, University of Eastern Piedmont, 28100 Novara, Italy

## Abstract

Myoepithelioma is an extremely rare tumour subtype and diagnosis is based on a wide variation of cellular morphology. FNAC specimens do not always suffice for a definitive differential diagnosis which depends on histology and immunohistochemistry of the lesion.* Case Presentation*. A 54-year-old female came to our attention with dysphagia and dyslalia of 6-month standing. Ear, Nose, and Throat (ENT) examination revealed a voluminous mass on the right portion of the base of her tongue, where postcontrast T2-weighted Magnetic Resonance Imaging (MRI) evidenced a hyperintense lesion. The fine-needle aspiration specimen taken for cytology was not diagnostic, as a differential diagnosis between myoepithelioma and a malignant neoplasm of the salivary glands necessitates parameters that cytology alone cannot provide. Therefore, the whole lesion was excised by diode laser through a transoral approach. Histology and immunohistochemistry of the completely excised lesion confirmed a myoepithelioma.

## 1. Introduction

Myoepithelioma is a rare benign myoepithelial tumour and its malignant counterpart is myoepithelial carcinoma. It is normally located in the major salivary glands; in fact myoepitheliomas arising from minor salivary glands are very rare, representing only 1.5% of all salivary gland tumours, and the base of the tongue is an unusual location [1, 2]. Myoepithelioma usually presents as an asymptomatic mass with a painless slow growth [1]. Imaging findings are nonspecific, and Magnetic Resonance Imaging (MRI) provides information strongly suggestive of a benign mass with a varying enhancement pattern. Various factors influence this variability such as histological component, stroma, vascularity, and histological cell type [3, 4]. Although cytology associated with immunohistochemistry could provide primary diagnostic information, it may not include the features whether it is malignant or not, leaving histological examination associated with immunohistochemistry of the surgical specimen as the gold standard for a definitive diagnosis. Recent interest has been placed in transmucosal Core Needle Biopsy (CNB) that can be of great help in submucosal space tumours of the oral cavity, especially when fine-needle aspiration cytology (FNAC) alone does not suffice. It requires, however, general anesthesia, experience of the surgeon, and a good exposure of the lesion, since a more cumbersome instrument is used [5]. Myoepithelial nature of neoplastic cells is obtained through a positive immunohistochemical reaction for muscle proteins [6]. Surgical excision with clear margins is the best treatment and selective neck dissection can be considered in case of patient with suspicious malignancy. Radiotherapy is reserved for lesions with surgical margins that are difficult to delineate [7].

## 2. Case Report

**Figure 1:**
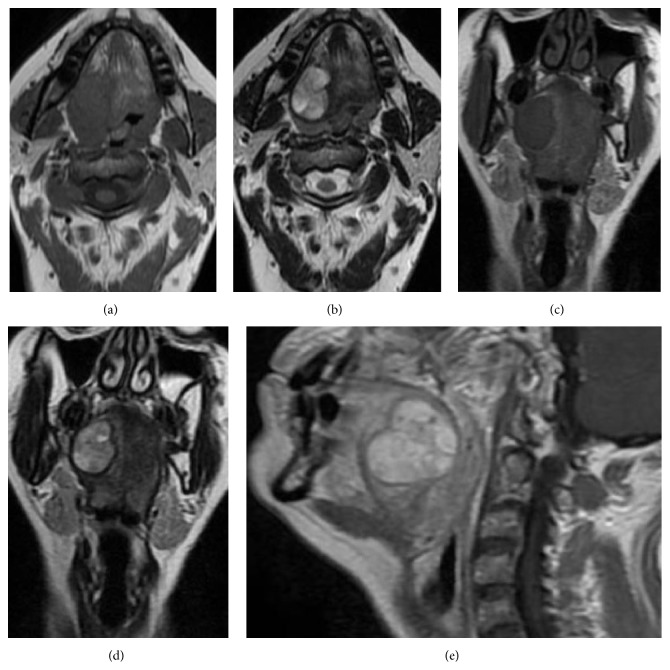
MRI images of axial section ((a-b), T1 and T2 weight) and coronal sections ((c-d), T1 and T2 weight); MRI images of sagittal section (e) showed the enhancing of the tumour mass involving the tongue base after gadolinium administration in T1 weight.

**Figure 2:**
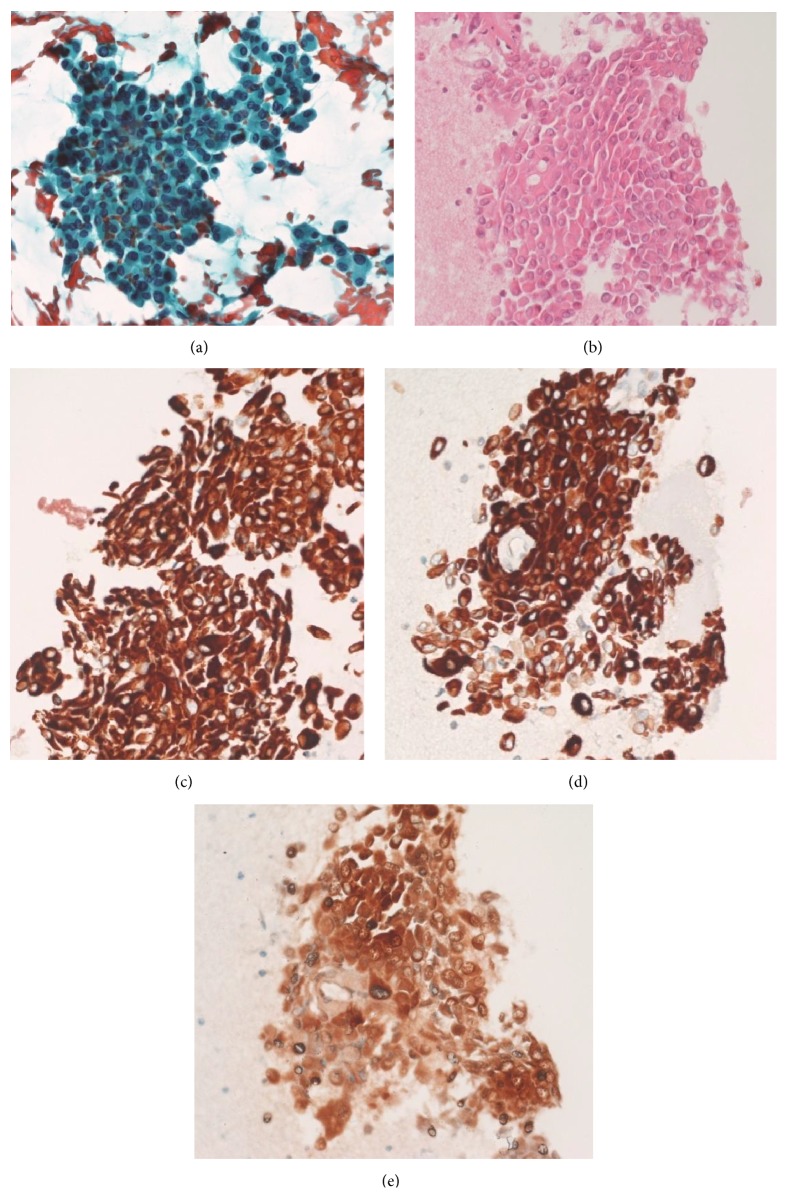
Cytological smears ((a), Papanicolaou staining) and histological sections from cytoinclusion ((b), H&E staining) showed nests of epithelioid cells, strongly positive for anti-pan-cytokeratin (c), anti-HMWCK, and S-100 (e) antibodies ((a–e): magnification 400x).

**Figure 3:**
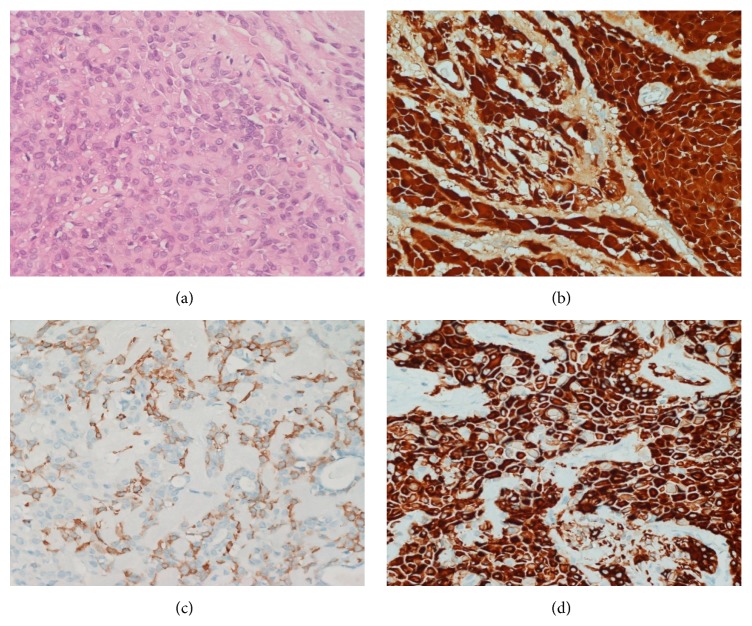
Histological specimens showing solid nests of plasmacytoid cells surrounded by thin fibrous stroma ((a), H&E staining) displaying diffuse immunoreactivity for S-100 (b) and focally for *α*-smooth muscle actin (c) and CK7 (d) ((a–d): magnification 400x).

A 54-year-old woman was referred to the Department of ENT of the Novara Hospital in February 2014, with a firm, painless, nontender mass involving the base of her tongue. The patient had been suffering from dysphagia, dyslalia, and sensation of an oropharyngeal foreign body for 6 months. Neither her clinical nor family history revealed personal or familiar cases of tumour and she was never a smoker. Extraoral examination revealed no abnormalities. Intraoral palpation revealed a nontender mass on the right side of the base of her tongue of approximately 3 × 3.5 cm, covered by normal oral mucosa. She reported stomatolalia and a deficit in tongue protrusion with deviation towards the right. MRI evidenced a capsulated, solid oval mass, with well-defined margins, of 31 × 28 × 21 mm on T1-weighted images without contrast; it was hyperintense and slightly inhomogeneous on the T2-weighted images, while T1 weighted images with contrast (gadolinium) showed remarkable enhancement of the mass (Figure 1). Fine-needle aspiration cytology (FNAC) was performed and the cytological specimen contained epithelioid cells, which were either isolated or aggregated in nests, which, at times, took on a three-dimensional appearance. Immunohistochemistry was positive for pan-cytokeratin, high molecular weight cytokeratin (HMWCK), and S-100 and negative for *α*-smooth muscle actin (Figure 2). A Core Needle Biopsy (CNB) was not performed due to the difficult exposure of the tumour, and as there was suspicion of a low grade malignant tumour of the salivary gland, supported by the presence of paralysis of the ipsilateral hypoglossal nerve, the patient was referred to surgery. A transoral partial glossectomy was performed using a diode laser under general anesthesia and the whole tumour was successfully excised. Ipsilateral lymph-node dissection of levels I–III, according to the Robbins classification, was carried out. Temporary tracheostomy ensured the patency of the airways, preventing an impairment of patient's breathing in the event of postoperative bleeding or oedema of the base of her tongue. The postoperative course was unremarkable and tracheostomy tube was removed at 6 postoperative days. Gross examination of the tumour showed a well-demarcated, oval elastic-hard mass, measuring 3 × 2.9 cm with a multilobular architecture and microcystic spaces filled with a clear fluid. At light microscopy, the H&E stained sections showed a neoplasm made up of plasmacytoid cells arranged in solid nests, with microcystic spaces and surrounding myxoid stroma or thin fibrous septa, displaying strong and diffuse immunoreactivity for S-100 protein and focally for *α*-smooth muscle actin, CK7, and p63 (Figure 3). These findings allowed for a definitive histological diagnosis of myoepithelioma to be made. There was no recurrence during the 18-month follow-up period.

## 3. Discussion

Myoepithelioma and its malignant counterpart, myoepithelial carcinoma, of the salivary glands are neoplasms made up almost exclusively of tumour cells with myoepithelial differentiation [6]. Their differential diagnosis includes a wide range of both benign and malignant tumours, depending on the predominant cell type: epithelioid, spindle, hyaline, clear cell [8]. In particular, the main differential diagnosis of myoepithelioma is pleomorphic adenoma (PA) [3, 9]. The distinction between the two is based on the fact that PA contains abundant ducts, but no more than one duct every medium to high power field or no more than one small cluster of the duct. Moreover, a much greater amount of stromal component is found in the PA compared to myoepitheliomas [9]. However, myoepithelioma is made up of a variety of plasmacytoid, epithelioid, and spindle-shaped cells with focally abundant mucoid stroma and may easily be confused with a PA. Despite the cytological differences, FNAC specimens do not always suffice for a definitive differential diagnosis [6]. Fortunately, all these tumours lack the immunohistochemical characteristics of myoepithelial cells, making immunohistochemistry of both cytological and surgical preparations crucial in definite diagnosis. The immunohistochemical criteria for myoepithelial differentiation are a double positivity for cytokeratins (in particular CK7 and CK14) and one or more myoepithelial markers, especially the S-100 protein, calponin, vimentin, and *α*-smooth muscle actin (SMA). Although most myoepitheliomas are biologically benign, malignant transformation of epithelioid myoepithelioma has been recently described; moreover, it occasionally infiltrates locally and metastasizes. Histological examination of the surgical specimen is still the gold standard to reach a definitive diagnosis of malignancy, due to the fact that it is based primarily on its infiltrative growth and, as cytological atypia may be absent in carcinomas, cytology alone does not suffice. In these cases, transmucosal Core Needle Biopsy (CNB) can be of great help for diagnosis but requires general anesthesia, experience of the surgeon, and a good exposure of the lesion [5]. The presence of a multinodular architecture with a hypercellular periphery, necrosis, and a significantly high mitotic count (ki-67 index) is useful to help recognise myoepithelial carcinomas [10]. It is advisable to perform a complete resection along with the surrounding salivary tissue to avoid recurrence [3]. We performed a surgical excision with tumour-free margins. The tumour was located on the base of the tongue, so an ipsilateral lymph-node dissection was made at levels I, II, and III. The data obtained confirm that conservative transoral surgery is feasible in this type of lesion and the prognosis appears to be good when surgical excision is complete. Close and prolonged follow-up is recommended.
